# Methamphetamine alters T cell cycle entry and progression: role in immune dysfunction

**DOI:** 10.1038/s41420-018-0045-6

**Published:** 2018-03-19

**Authors:** Raghava Potula, Bijayesh Haldar, Jonathan M Cenna, Uma Sriram, Shongshan Fan

**Affiliations:** 1 Department of Pathology and Laboratory Medicine, Lewis Katz School of Medicine, Temple University, Philadelphia, PA 19122 USA; 2 Center for Substance Abuse Research, Lewis Katz School of Medicine, Temple University, Philadelphia, PA 19122 USA

## Abstract

We and others have demonstrated that stimulants such as methamphetamine (METH) exerts immunosuppressive effects on the host’s innate and adaptive immune systems and has profound immunological implications. Evaluation of the mechanisms responsible for T-cell immune dysregulation may lead to ways of regulating immune homeostasis during stimulant use. Here we evaluated the effects of METH on T cell cycle entry and progression following activation. Kinetic analyses of cell cycle progression of T-cell subsets exposed to METH demonstrated protracted G1/S phase transition and differentially regulated genes responsible for cell cycle regulation. This result was supported by in vivo studies where mice exposed to METH had altered G1 cell cycle phase and impaired T-cell proliferation. In addition, T cells subsets exposed to METH had significant decreased expression of cyclin E, CDK2 and transcription factor E2F1 expression. Overall, our results indicate that METH exposure results in altered T cell cycle entry and progression. Our findings suggest that disruption of cell cycle machinery due to METH may limit T-cell proliferation essential for mounting an effective adaptive immune response and thus may strongly contribute to deleterious effect on immune system.

## Introduction

A commonly abused drug worldwide, methamphetamine (METH) in past two decades has become a major public health and safety problem^1^. A potent central nervous system (CNS) stimulant that induces the release of biogenic amines from nerve terminal, METH is extremely addictive and has deleterious effects on immune system^2–10^. We along with other recent studies have demonstrated the METH effects on both innate and adaptive immune system^1,7,9,11^, including inhibition of antigen presentation, impairment of phagocytosis^2,12^, altered gene expression of immune cells^5^. The alkalizing ability of METH has been thought to possibly result in cellular dysfunction, where organelles within immune cell are normally acidic. Induction of IL-4 and IL-10 cytokines known to inhibit T-cell proliferation ^2^, suppression of Th1 cytokine (IL-2 and IFN-γ) and increased TNF-α production^7^ have been reported in animal upon METH exposure.

The ability of lymphocytes to proliferate and differentiate into effector cells in response to antigenic stimuli is essential for generation of a robust adaptive immune response^13^. Previous studies have shown that METH exerts immunosuppressive effects on antigen-presenting cells (APC), including dendritic cells and macrophages^6,7,12^. Most recent evidence for disruption of immune homeostasis in METH administered mice elucidate specific cellular alterations induced by METH on key subsets of leukocytes^14^. Coherent with the understanding that T-cell proliferation in response to a stimulus is an appropriate indicator for cellular immunity, we have reported earlier that METH results in the loss of T-cell proliferative activity^15^. Cell cycle regulators play a fundamental role in controlling lymphocyte proliferation^16,17^. Cyclins, the key elements of cell cycle progression machinery, and their associated cyclin-dependent kinases (CDKs) play an important role in cell cycle transition and regulation^16,17^. It is generally accepted that suboptimal T effector function in response to antigen presentation is characterized by low IL-2 production and cell cycle arrest at the G1/S phase^7^. Activation of cell induces the expression of the D-type cyclins that activates CDK4 and/or CDK6, prompting entrance into G1 phase^16^. Activation of E2F mediates transcription of genes responsible to move cell into S phase^16,17^. Cyclin E/CDK2 complexes regulate transition from G1 to S phase; the cyclin B/CDK1 complex regulates transition from S to G2 phase. Given that the ability to regulate both cell cycle progression and proliferation is central to the maintenance of immune homeostasis, in the present study, we sought to examine the effects of METH on T cell cycle entry and progression. Our findings show that METH exposure creates a cellular environment that potentiates impairment of cell cycle machinery, owing to the limited proliferative potential of the T-cell subsets. Alternation of cell cycle machinery due to METH might have broader implication contributing to the suppressed immune response that come in play in response to chronic viral infection such as HIV-1.

## Results

### T cell cycle transcriptional network is differentially regulated by METH

Previously, work in our lab has shown that METH exposure results in the loss of T-cell proliferative activity^15^. Dynamic changes in the cell cycle pathway gene expression regulate the specific CDK activities as a function of cell cycle and proliferation. To further investigate our previous findings and gain new insights into the effects of METH on cell cycle exit and progression of T lymphocytes, we performed cell cycle gene expression profile of human pan T stimulated with anti-CD3/CD28 in the absence or presence of METH (100 μM) using a Human T cell cycle RT² Profiler™ PCR array. mRNA expression levels of 84 genes known to be involved in the various interphases of the cell cycle in CD4+ and CD8+ T cells subsets of METH treated, and controls were compared. Details of the genes function and their fold changes in METH treated T-cell subsets compared to control are shown in Table 1.

**Table 1:** Differential transcription profile of cell cycle pathway genes in METH treated T cells

Gene symbol	Description	Physiological role	Fold change
G1 phase and G1/S transition			
*CDKN3*	Cyclin-dependent kinase inhibitor 3	Cell cycle regulation: Dephosphorylates CDK2 in a cyclin-dependent manner.	2.00↑
*CUL1*	Cullin 1	Ubiquitination of proteins involved in cell cycle progression, signal transduction and transcription.	1.98 ↓
S phase and DNA replication			
*ABL1*	C-abl oncogene 1, receptor tyrosine kinase	Regulation of cytoskeleton remodeling during cell differentiation, cell division and cell adhesion.	2.01↑
*MCM2*	Minichromosome maintenance complex component 2	Role in the initiation of DNA replication, replication fork movement, and intimately related to cell proliferation.	2.02 ↓
*MCM4*	Minichromosome maintenance complex component 4	Central role in the regulation of DNA replication	2.03 ↓
G2 phase and G2/M transition			
*CCNB1*	Cyclin B1	Essential for the control of the cell cycle at the G2/M (mitosis) transition	2.01↑
*CCNG1*	Cyclin G1	Modulator of the cell cycle and apoptosis, transcriptional target of p53 tumor suppressor gene	1.94 ↓
*CCNT2*	Cyclin T2	Regulatory subunit of the cyclin-dependent kinase pair essential for the elongation of transcription and cotranscriptional processing by RNA polymerase II	1.98↑
*CDK5R1*	Cyclin-dependent kinase 5, regulatory subunit 1 (p35)	Regulatory subunit of the cyclin-dependent kinase pair (CDK9/cyclin-T1) complex P-TEFb.	2.02↑
Cell cycle checkpoint, arrest and regulation			
*CCNG2*	Cyclin G2	Role in growth regulation and in negative regulation of cell cycle.	2.32↑
*RB1*	Retinoblastoma 1	Negative regulator of the cell cycle, acts as a transcription repressor of E2F1 target genes.	1.99↑
*RBBP8*	Retinoblastoma binding protein 8	Regulates cell proliferation and complexes with transcriptional co-repressor CTBP.	1.99 ↓
*CCND2*	Cyclin D2	Control of the cell cycle at the G1/S (start) transition.	2.00 ↓
*BCL2*	B-cell CLL/lymphoma 2	Regulator of programmed cell death or apoptosis of cells such as lymphocytes by controlling the mitochondrial membrane permeability.	1.99↓
*TFDP1*	Transcription factor Dp-1	Control of cell-cycle progression from G1 to S phase	2.02↑

### METH alters cell cycle progression of CD3/CD28 activated T cells

**Fig. 1:**
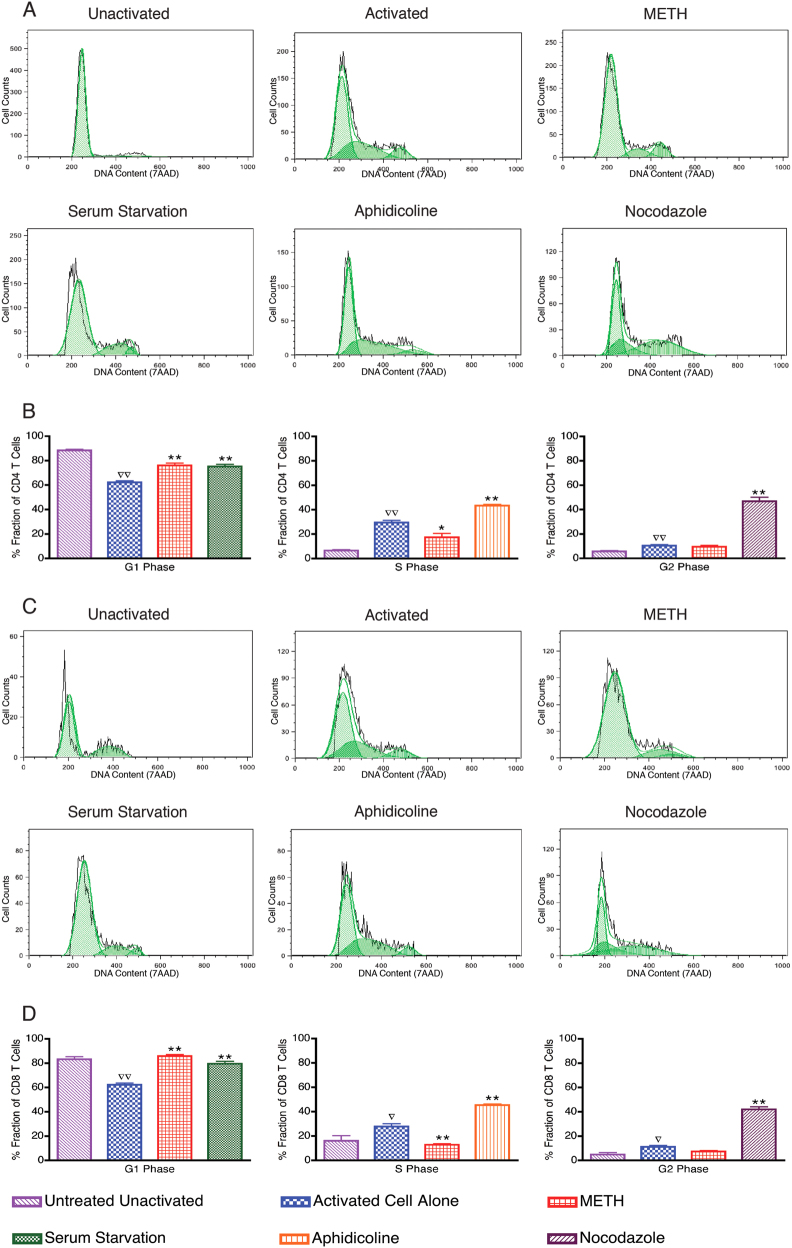
METH alters cell cycle progression of CD4+ / CD8+ T cells. PBMCs were stimulated with human anti-CD3/CD28 for 48 h to induce proliferation. After activation, PBMCs were either serum starved or treated with METH (100 μM), Aphidicoline (100 ng/ml) or Nocodazole (25 μM) for 24 h. The cells were stained with 7AAD and cell cycle data analysis was performed on CD4+ and CD8+ population. FlowJo Dean-Jett-Fox (DJF) cell cycle modeling algorithm was used to define the G1, S and G2 phases of the cell cycle. The data are presented as cellular DNA content frequency histograms. The green overlay indicates the fit curves to the stages of the cell cycle. Results are representative of nine independent experiments from five separate donors. **a** Representative DNA content frequency histograms of CD4+ T cells. **b** The data from the cell cycle distribution of CD4+ T cells are summarized and presented as the mean ± SEM of nine independent experiments. **c** Representative DNA content frequency histograms of CD8+ T cells. **d** The data from the cell cycle distribution of CD8+ T cells are summarized and presented as the mean ± SEM of nine independent experiments. ∇*P* < 0.05 and ∇∇*P* < 0.001 compared to unactivated cells; *P < 0.05 and **P < 0.001 compared to activated cells

Cell cycle regulators play a fundamental role in controlling lymphocyte proliferation. To evaluate the effects of METH on co-stimulatory-driven T cell-cycle entery and progression, PBMC were cultured with anti-CD3/CD28 with and without METH. Cell cycle interphase was determined by cell synchronization studies. METH-treated cells were synchronized with cells either serum starved, or treated with aphidicolin (100 ng/ml) and nocodazole (25 μM) for 24 h to determine G1, S or G2 interphase, respectively. Figure 1a depicts a representative DNA content (7AAD) histogram of CD4 + T cells within phases of cell cycle in response to the indicated stimuli. As expected majority of CD4+ T cells from G0/G1 phase at resting after co-stimulation entered S phage followed by G2 phase (Fig. 1b). Interestingly, the % fraction of CD4+ T cells in the G1 phase of METH treated cells following co-stimulation were significantly (*P* < 0.001) higher compared to G1 phase of untreated stimulated cells (Fig. 1b). We also found the % fraction of CD4 + T cells in METH-treated cells in the S phase was significantly (*P* < 0.05)lower compared to control-stimulated cells (Fig. 1b). However, in the G2 phase, the % fraction of CD4+ T cells in METH-treated cells were similar to control-stimulated cells (Fig. 1b).

The pattern of cell cycle of CD8+ T subset cells in METH treated cells mirrored CD4+ T cells. Figure 1c depicts a representative DNA content (7AAD) histogram of CD8+ T cells found within phases of cell cycle in response to the indicated stimuli. After treatment with METH for 24 h, the % fraction of CD8+ T cells in METH-treated cells following co-stimulation were significantly (*P* < 0.001) higher to G1 phase and lower (*P* < 0.001) in S phase compared to control-stimulated cells (Fig. 1d). There was no difference in % fraction of CD8+ T cells in the G2 phase in the control-stimulated cells and METH-treated cells. Taken together, these results indicate that METH protracts the G1/S phase transition, and the S phase entry in METH-treated cells.

### METH blocks upregulation of cyclin E expression of CD3/CD28 activated T cells

**Fig. 2:**
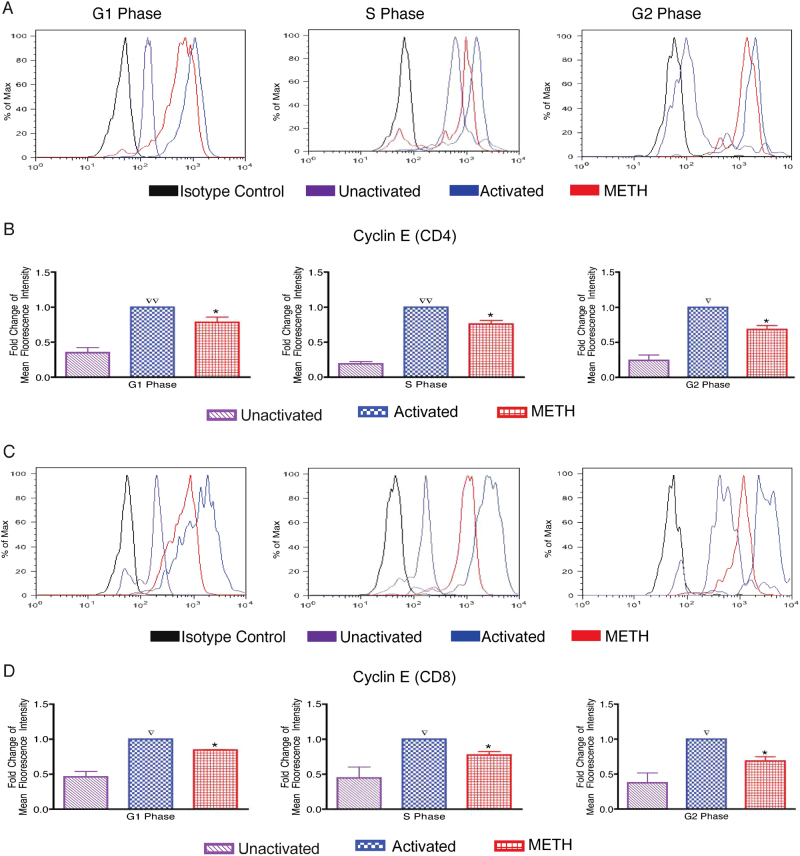
Suppressed cyclin E expression of CD4/CD8+ T cells after METH treatment. PBMCs were stimulated with human anti-CD3/CD28 for 48 h and then treated with METH (100 μM) for 24 h. **a** Representative histogram of fluorescence intensity of CD4+ T cells. **b** Fold change of Cyclin E expression in METH treated cells in respective phase in total gated CD4+ T-cell population. **c** Representative histogram of fluorescence intensity of CD8+ T cells. **d** Fold change of Cyclin E expression in METH treated cells in respective phase in total gated CD8+ T-cell population. The data from the cyclin E expression were summarized and presented as the mean ± SEM of five independent experiments. ∇*P* < 0.05 and ∇∇*P* < 0.001 compared to unactivated cells; *P < 0.05 compared to activated cells

To understand the underlying basis of altered cell cycle progression in CD4+ and CD8+ T subset of METH-treated cells, we studied the expression of cell-cycle regulatory proteins involved in the G1 transition and S phase entry of anti-CD3/CD28-costimulated human resting T cells. During G1/S phase progression; cyclins D (D1–D3) act in mid-G1, followed by cyclin E and cyclin A involved at the G1/S boundary. Cells cultured with anti-CD3/CD28 resulted in induction of D, A and B cyclins, however, the expression of these cyclins were not affected by METH (data not shown). Figure 2a shows representative histogram of cyclin E fluorescence intensity of CD4+ T cells found within phases of cell cycle in response to the indicated stimuli. Induction of cyclin E expression was significantly impaired by METH; 1.3-fold in G1 (*p* value 0.047), 1.3-fold in S (*p* value 0.0175) and 1.5-fold in G2 (*p* value 0.031) phase as compared to the respective untreated activated cells (Fig. 2b). Figure 2c is the representative histogram of cyclin E fluorescence intensity of CD8+ T cells, demonstrating similar fold reduction of cyclin E expression in METH treated cells; 1.2-fold in G1 (*p* value 0.02), 1.3-fold in S (*p* value 0.009) and 1.5-fold in G2 (*p* value 0.007) phase as compared to the respective untreated activated cells (Fig. 2d). Taken together, our data suggest that METH treatment renders suppression of cyclin E expression in both T cells subsets.

### METH impairs CDK2 expression in CD3/CD28-stimulated T cells

**Fig. 3:**
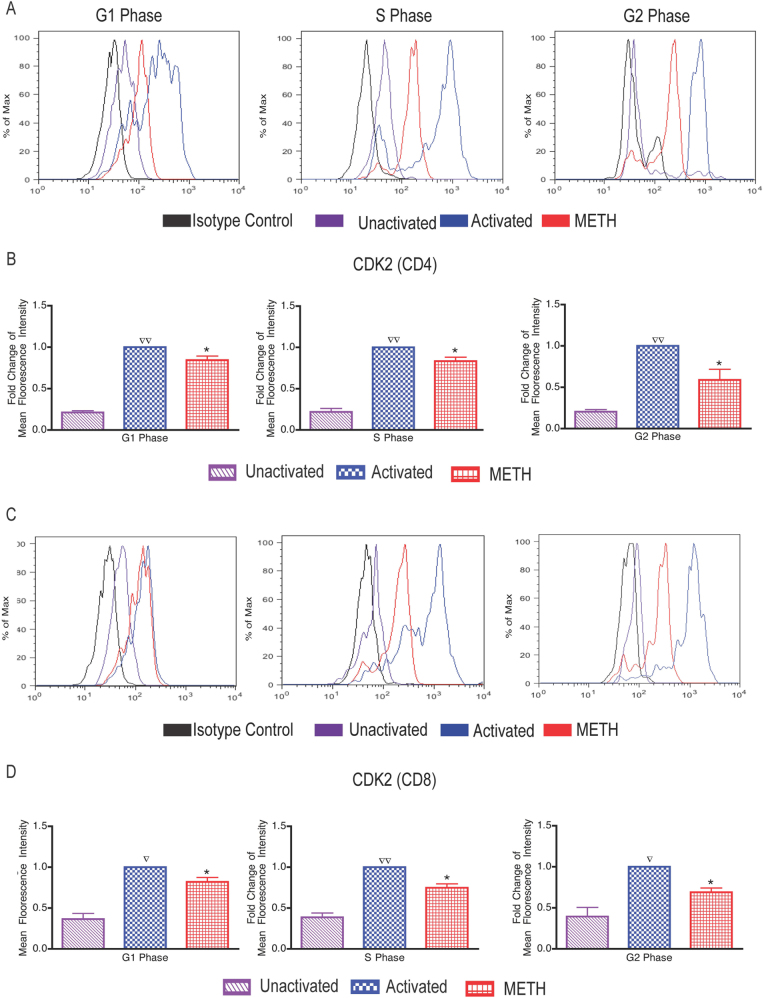
Impaired CDK2 expression of CD4/CD8+ T cells following METH treatment. PBMCs were exposed to METH (100 μM) for 24 h following stimulation with human anti-CD3/CD28 for 48 h. **a** Representative histogram of fluorescence intensity of CD4+ T cells. **b** Fold change of CDK2 expression in METH treated cells in respective phase in total gated CD4+ T-cell population. **c** Representative histogram of fluorescence intensity of CD8+ T cells. **d** Fold change of CDK2 expression in METH treated cells in respective phase in total gated CD8+ T-cell population. The data from the CDK2 expression were summarized and presented as the mean ± SEM of six independent experiments. ∇*P* < 0.05 and ∇∇*P* < 0.001 compared to unactivated cells; *P < 0.05 compared to activated cells

Activation of CDK2 by cyclin E subsequently allows cells to move into S phase. Since cyclin E is known to bind CDK2 and our results showed low cyclin E activity in the presence of METH, we next examined the CDK2 activity in activated T cells in presence of METH. Figure 3a is the representative histogram of CDK2 fluorescence intensity and Fig. 3b shows the fold change in CDK2 expression of CD4 + T cells. The level of CDK2 expression in METH treated was significantly decreased by 1.2-fold in G1 (*p* value 0.02), 1.2-fold in S (*p* value 0.02) and 1.7-fold in G2 (*p* value 0.03) phase as compared to the control cells in respective cell cycle stages of interphase (Fig. 3b). METH affected activated CD8 + T cells in similar manner (Fig. 3c, d), CDK2 expression in METH treated was decreased by 1.3-fold in G1 (*p* value 0.02), 1.3-fold in S (*p* value 0.01) and 1.7-fold in G2 (*p* value 0.05) phase as compared to the corresponding cell cycle phase in controls stimulated cells (Fig. 3d).

### METH alters E2F1 expression in T cells independent of retinoblastoma tumor suppressor protein (pRB)

**Fig. 4:**
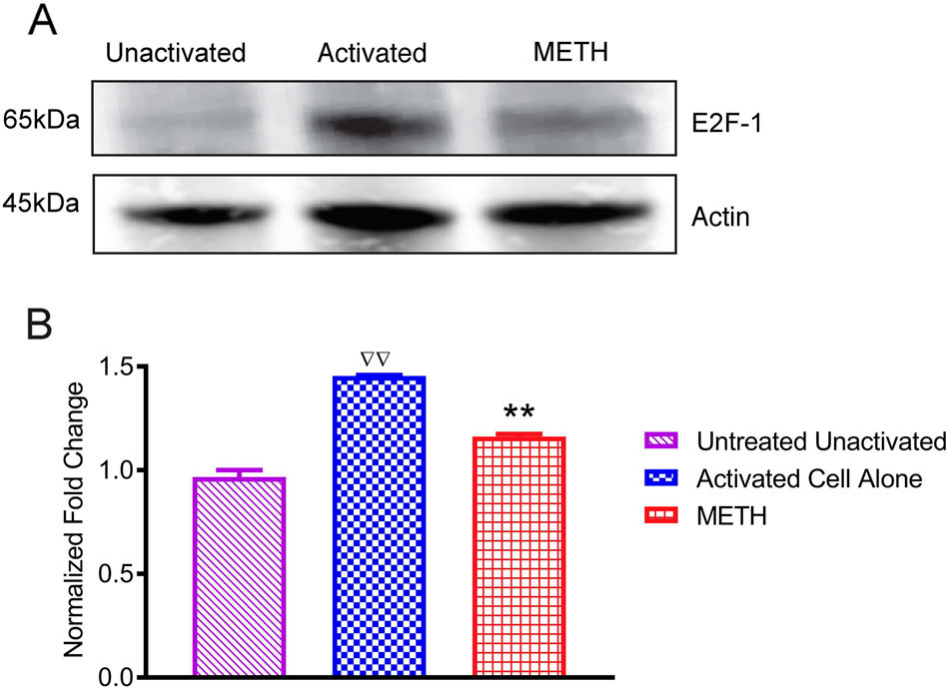
Decreased expression of E2F1 protein in T cells in response to METH. Primary human T were activated with human anti-CD3/CD28 for 48 h treated with METH (100 μM) for 24 h. **a** Representative immunoblots of E2F1 and internal standard actin of three independent experiments are shown in blots. **b** The relative fold expression of protein subunits normalized to actin are shown in the histogram. The data are presented as the mean ± SEM of three independent experiments. ∇∇*P* < 0.001 compared to unactivated cells;**P < 0.001 compared to activated cells

The feedback loops in the pRB–E2F pathway enable both the regulation and the fine-tuning of E2F, which is crucial to its proper and timely activity. Since the E2F family of transcription factors is considered to have a pivotal role in controlling cell-cycle progression we investigated the expression of E2F1 after METH treatment to elucidate the mechanism behind METH-induced delay of G1-S transition. As seen in Fig. 4a, b, immunoblot analysis of protein in whole cell lysate obtained from T cells treated with METH revealed significant decrease in E2F1 expression as compare to control T cells (*p* value < 0.001). Activation of CDK2 by cyclin E initiates phosphorylation of pRB leading to activation of E2F-mediated transcription, allowing cells to move into S phase. Therefore, we tested whether the reduced level of CDK2 and cyclin E has an impact on pRB phosphorylation. Interestingly, no change in pRB phosphorylation was observed in either of the T-cell subsets of METH treated cells compared to controls (data not shown).

### METH suppresses cell proliferation in vivo

**Fig. 5:**
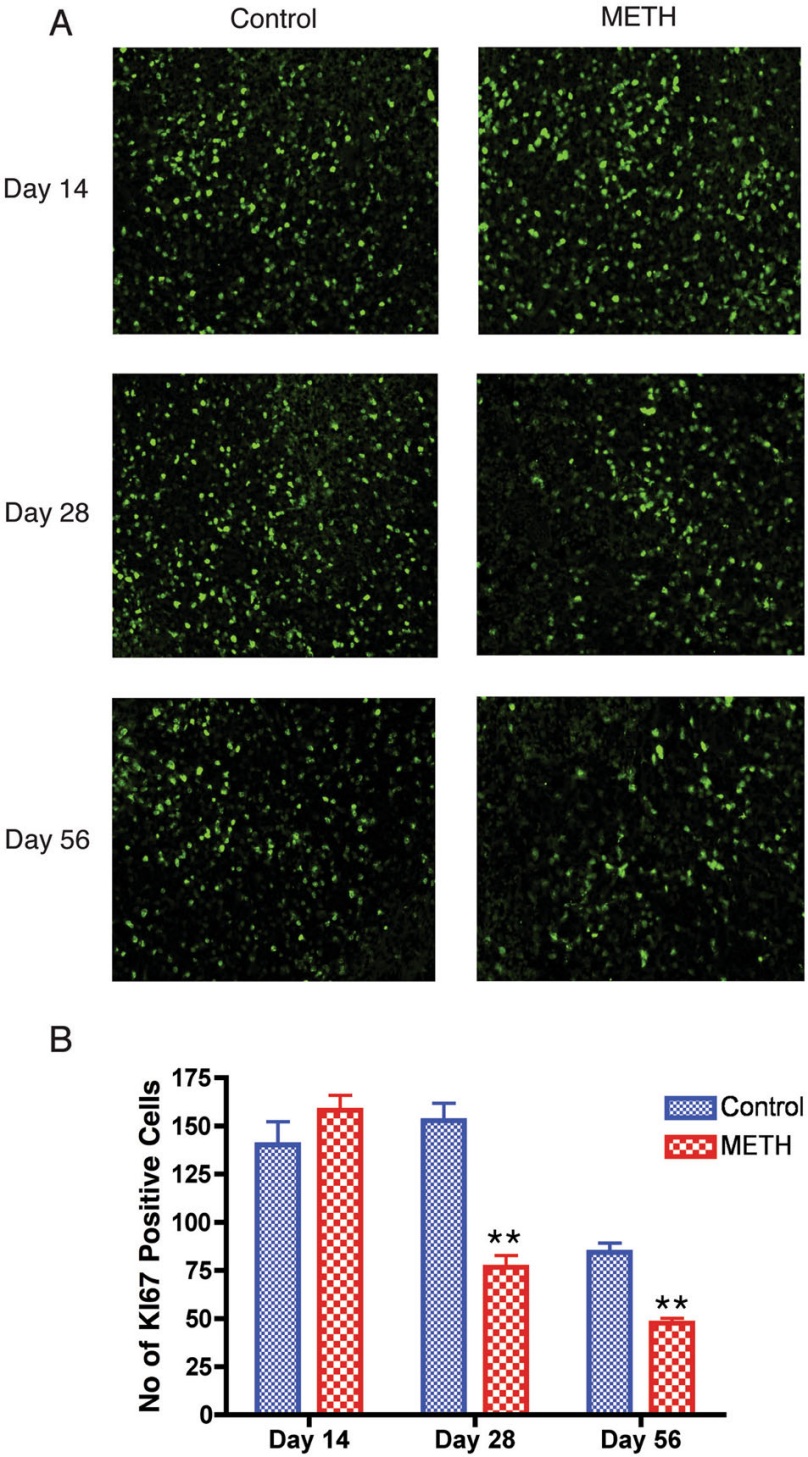
Methamphetamine alters numbers of proliferating cells of mouse spleen. Paraffin-embedded spleen was sectioned at 5 μm and immunostained with anti-Ki67 antibody. (A) Representative images of control and METH mouse spleens immunostained with anti-Ki67 antibody. (B) Data representing semiquantitative analyses of Ki67 staining from five different field for each mice (n = 3) presented as the mean ± SEM. **P < 0.001 compared to control mice

**Fig. 6:**
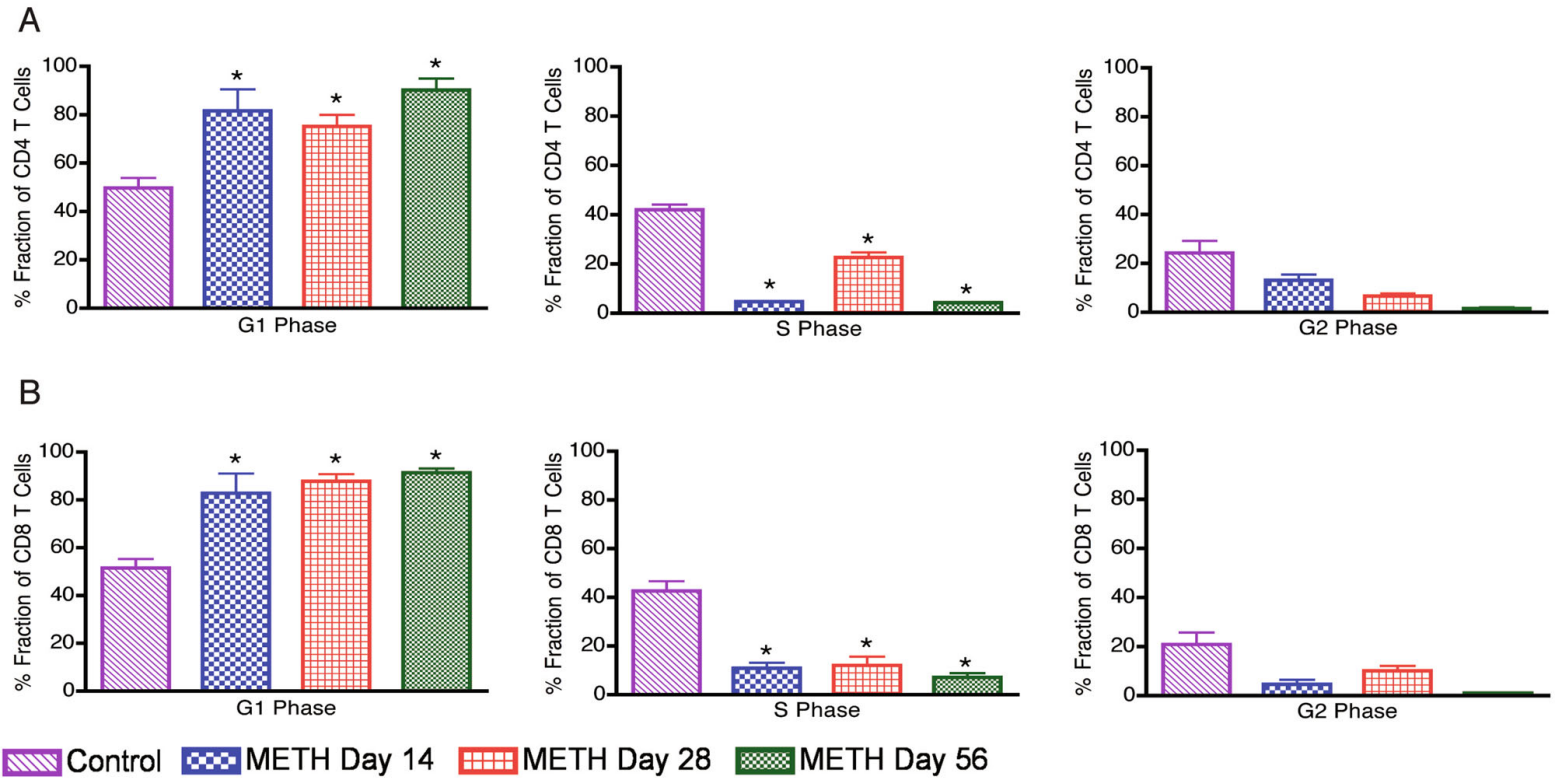
Impaired cell cycle progression of CD4+ and CD8+ T cells in mice exposed to METH. Splenocytes were stimulated with mouse anti-CD3/CD28 for 48 h to induce proliferation. After activation cells were stained with 7AAD and cell cycle data analysis was performed on CD4+ and CD8+ population. FlowJo Dean-Jett-Fox (DJF) cell cycle modeling algorithm was used to define the G1, S and G2 phases of the cell cycle. Results are representative of two different experiments from three mice per day. **a** Average values of % fraction of cells in respective phase in total gated CD4+ T-cell population. **b** Average values of % fraction of cells in respective phase in total gated CD8+ T-cell population. *P value < 0.05 compare to control mice

To investigate the effect of METH on cell cycle progression in vivo, we performed immunohistochemical analysis of Ki-67 protein expression, most commonly used as a proliferation markers^18^. Figure 5a shows representative pictures of Ki-67 staining of control and METH-treated mouse spleen sections. Quantitative analysis was performed by determining the number of Ki-67 positive cells in five representative microscopic fields for each tissue section. The data are represented as average number of Ki67-positive cells for control and METH treated mice (Fig. 5b). Mice treated with METH at days 28 and 56 showed significantly (*p* value < 0.001) less Ki-67 positive cells compared to controls, indicating that less percentage of cells were proliferating under METH treatment. This may be attributed to the observations that METH treatment results in altered cell cycle progression in vitro (Fig. 1b, d). To further assess the suppression of cell proliferation, cell cycle pattern of splenocytes from METH-treated mice were compared to that of the control mice. As shown in Fig. 6, the percent fraction of CD4+ and CD8+ T cells in different cell cycle interphase stages was calculated as described in “Materials and Methods” section. Splenocytes from METH-treated mice had significantly higher number of CD4+ T cells in G1 phase and lower in S phase at 14, 28 and 56 days when compared with from splenocytes from control mice (Fig. 6a). Consistent with this observation, the cell cycle phase distribution of CD8+ T cells was found to be similar in the splenocytes of METH-treated mice compared to untreated mice (Fig. 6b). Collectively, these data demonstrate that METH treatment impedes proliferation in vivo and altered G1/S phase transition of T cell cycle consistent with the *in vitro* studies.

## Discussion

The highly addictive stimulant, METH is abused by millions and known to alter immune function and increase susceptibility to infection. Although the immunomodulatory effects^2,4,6,7,19^ of METH have been extensively investigated, the underlying mechanisms involved in the deregulation of acquired immune response, in particular T-cell responses is still unclear. The ability of T cells to respond efficiently is crucial to most adaptive immune responses against foreign antigens. In this regard, our earlier study has shown that exposure of T cells to METH results in the loss of T-cell proliferative activity^15^. Of relevance are our recent findings in a murine model that suggests, METH-induced microenvironment results in upregulation of immunoinhibitory programmed cell death-1 (PD-1) marker, alters homeostatic proliferation and differentiation pathways of T-cell subsets in an animal model of chronic viral infection^20,21^. In this present study, we explored the effect of METH on cell cycle regulators and their role in suppression of T-cell proliferation. Genes regulated during the cell cycle encode several proteins that function in the subsequent phase of the cell cycle. Therefore, we profiled gene expression pattern of 84 genes that regulate cell cycle proliferation of primary human T lymphocytes (Table 1). The observed differentially regulated gene included functional phenotypes of genes that; positively and negatively regulate the cell cycle, genes associated with transitions between the each of the phases, DNA replication, checkpoints and arrest. The altered gene expression level suggests that the expression level in METH-treated primary human T cells could probably modulate expression of cyclins thereby regulate CDK activity and progression of cell cycle.

Chronic exposure to METH has been shown to result in long-term disruption of the cell cycle phases in endothelial cells^22^ and alters astrocytes normal progression by inducing cell cycle arrest^23^. Therefore, to further assess the METH-induced dynamic changes in the gene expression as a function of cell cycle progression, we performed time-lapse measurements of cell populations synchronized in the cycle by the agent arresting them at a specific point of the cycle. Flow cytometric analysis was used to monitor the progression of cells through cell cycle phases and subsequently investigate the cell cycle-regulated molecules. A crucial regulatory point controlling the onset and tempo of cell division is the transition from the G1 into the S phase^24,25^. Interestingly, in contrast to controls, METH-exposed T-cell subsets remained in the G0/G1 phase following stimulation by crosslinking anti-receptor antibodies (Fig. 1). In regards to this, the duration of time METH-exposed T cells are in the G0/G1 phase of the cell cycle probably greatly affects the vigor of cell proliferation, which we have previously reported^15^.

The intricate process of cell division is governed by complex multiple regulatory points through the entire process of cell cycle by transcriptional and epigenetic processes. A major processes governing the cell cycle progression through the G1/S border and the entry of cells into S phase of the cell cycle is CDK2–cyclin E complex. Cyclin E acts as a limiting factor for G1 phase progression and S phase entry ^26^. Suppression of cyclin E (Fig.2) by METH in both CD4 and CD8 T-cell subsets further highlights the effect of METH on T-cell proliferation and disruption of cell cycle. During the process of T-cell proliferation, enzymatic activity of the CDKs is expressed at elevated levels, thereby regulating the G1/S transition. The suppressed level of CDK2 expression in METH-treated cells (Fig. 3) highlights the role CDK2–cyclin E complex may play in influencing cell cycle progression under the influence of stimulant use.

Phosphorylation of the tumor suppressor Rb family proteins, one of the substrates of cyclin E-CDK complexes^27–29^ serve as critical regulators in the process of transition from G1 into S phase and the initiation of DNA synthesis. Dissociation of pRB by cyclin-CDK complexes during G1 phase from the transcription factor E2F triggers the process of transcription factor mediated initiation of target genes expression that are essential to drive a cell into S phase^26^. T cells exposed to METH had significant reduction in level of E2F1 at protein level as compared to control cells (Fig. 4). Interestingly, pRB hyperphosphorylation levels remained unchanged in METH-treated cells (data not shown) suggesting that the observed changes in E2F proteins expression in METH treated cells are probably due to mechanisms independent of pRB phosphorylation^30^. Since E2F proteins form a family of transcription factors that regulate the transition from the G1 to the S phase in the cell cycle, the decreased level of E2F1 after METH treatment may be critical for the control of cell cycle time and the duration of G1.

Expression of Ki-67 protein is associated^31^ with cell proliferation and is widely used in routine pathology as a “proliferation marker”. The Ki-67 protein is present during all active phases of the cell cycle (G1, S, G2, and mitosis), but is absent from resting cells (G0), making it an excellent marker for determining the growth fraction of a given cell population. In our current study, we found that mice that were injected with METH for 28 and 56 days had a significantly lower number of Ki67-positive cells as compare to control mice, suggesting that METH impairs the rate of cell proliferation in vivo. The impaired cell proliferation ultimately leads to impaired cell cycle progression. Both CD4+ and CD8+ T cells from METH injected mice splenocytes were found to be interrupted in G1 phase at day 14, 28 and 56, as compare to control mice splenocytes. This results further support the fact that METH treatment alter normal T cell cycle progression by disrupting the G1 phase of the cell cycle.

Our findings thus provide a new perspective for understanding the molecular mechanism underlying the aberrant T-cell immune functions observed upon METH exposure. In summary, following antigenic stimulation in response to METH, CD4+ and CD8+ T cell-cycle progression is disrupted in vitro. The aberrant rate T cell cycle progression is due to prolonged G1 phase. Mice exposed to METH manifest a defect in proliferation. Besides reduced proliferation, METH induces slowed cell-cycle kinetics, primarily due to the protracted G1 phase in both CD4+ and CD8+ splenocytes. In response to METH, CD4+ and CD8+ T cyclin E expression is suppressed in vitro. Additionally, the cyclin-dependent kinase, CDK2 expression and E2F1 expression is suppressed while one of the substrates of cyclin E-CDK complexes, pRB appear to be unaltered. Thus, our new findings suggest that METH exerts its immunomodulatory effects on T cells by suppressing the expression of cyclin E, CDK2 and E2F1, which are key regulators of normal cell cycle progression.

## Materials and methods

### Reagents and Antibodies

l-Methamphamphetamine Hydrochloride was purchased from Sigma-Aldrich (St. Louis, MO). Aphidicolin and Nocodazole were purchased from EMD Millipore (Billerica, MA). Optimal concentrations of these drugs were based upon preliminary dose-response experiments and did not affect cell viability. Alexa Fluor 647 mouse anti-human CD20, brilliant violet 421 mouse anti-human CD4 and CD8, mouse anti-human CD3 and CD28 antibodies were purchased from Biolegend (San Diego, CA). V500 rat anti-mouse CD4 and mouse anti-human CD8 antibody was purchased from Becton–Dickinson (Mountain View, CA). Rabbit anti-human CDK2 and E2F1 antibodies were purchased from Cell Signaling (Danvers, MA) and rabbit anti-human Cyclin E antibody was obtained from Novus Biologicals (Littleton, CO). The anti-rabbit IgG Alexa Fluor 647 conjugate secondary antibody and all isotype controls were purchased from Cell Signaling (Danvers, MA). APC-eFluor780 anti-mouse CD3 antibody and 7-amino-actinomycin D (7AAD) were purchased from eBioscience (San Diego, CA). Ki67 antibody was purchased from (Abcam, Cambridge, MA). ProLong Antifade plus DAPI (4’,6-diamidino-2-phenylindole) was purchased from Life Technologies, CA). Alexa-fluor 488-conjugated goat anti-rabbit secondary antibody was purchased from Life Technologies (California).

### Cells and treatment

Peripheral blood mononuclear cells (PBMC) were isolated from leukoreduction filters (Pall Corporation, Port Washington, NY) of normal blood donors obtained from American Red Cross within 2 to 6 h after blood collection as described previously^32–34^. Briefly, after collection, the filters were back-flushed with 50 ml phosphate-buffered saline (PBS) containing 5 mmol ethylenediaminetetraacetate (EDTA). PBMCs were then isolated using standard Ficoll density gradient separation. Cell concentration was adjusted to 1 × 10^6^/ml in R10 complete media [RPMI 1640 medium (CellGrow, Manassas, VA) supplemented with 10% heat-inactivated FBS (Life Technologies corporation, Carlsbad, CA), 2 mM l-glutamine (Life Technologies corporation, Carlsbad, CA), 100 U/ml penicillin (Life Technologies corporation, Carlsbad, CA), 100 μg/ml streptomycin (Life Technologies corporation, Carlsbad, CA) and 20 mM HEPES (Life Technologies corporation, Carlsbad, CA). For TCR/CD28 co-stimulation, PBMCs or mouse splenocytes were incubated with 10 μg/ml and 4 μg/ml of soluble CD3 and CD28 antibodies respectively for 48 h. For cell synchronization experiments, PBMCs were either serum starved, treated with Aphidicolin (100 ng/ml) or Nocodazole (25 μM) (to synchronize the cells in G1, S and G2 Phase, respectively) or treated with METH (100 μM) for 24 h. After 24 h of serum starvation or treatment with specified drug, PBMCs were kept in complete media for 6 h for recovery before surface staining for CD4 and CD8. For some experiments, pan-T cells were obtained directly from the Human Immunology Core facility of the University of Pennsylvania and cultured as described perilously^15^. Single-cell suspensions of mouse splenocytes were prepared by standard gentle mechanical disruption through a 70 μm nylon mesh screen (BD Falcon, Mountain View, CA) using a 3 ml syringe plunger (BD Biosciences, Mountain View, CA), followed by removal of red blood cells with RBC Lysis Buffer Solution (eBioscience, San Diego, CA).

### RT² profiler PCR array

Total RNA from T cells was purified by using the RNeasy mini kit (QIAGEN, Valencia, CA) according to the manufacturer’s manual. The effects of METH on the expression of 84 cell cycle-regulated genes were examined using human cell cycle RT^2^ Profiler PCR array (SABioscience, Valencia, CA) according to the manufacturer’s instructions. Data analysis was performed using the δδCt method with the aid of an analysis template provided by the SuperArray website (http://www.superarray.com/manuals/pcrarraydataanalysis.xls). The results are expressed as the fold change in gene expression from the control group.

### Flow Cytometry

**Fig. 7:**
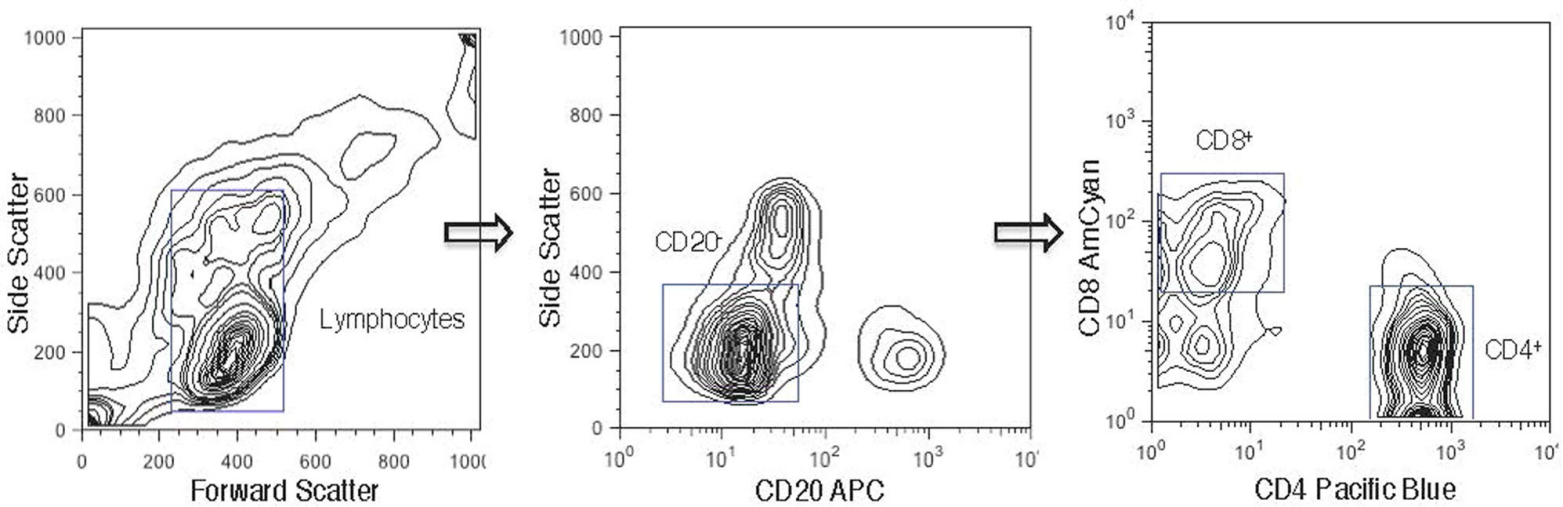
Gating strategies for the flow cytometry analyses of peripheral blood leukocytes. Peripheral blood leukocytes from healthy individuals (*n* = 5) were separated into two broad subsets following staining with cell surface marker CD20, using flow cytometry. Based on their distribution in a side scatter (SS) vs. forward scatter (FS) contour plot, viable leukocytes were selected. B lymphocytes were identified based on their binding of the CD20 antibody, while CD20-negative lymphocytes were identified as T lymphocytes. From this CD20-negative T lymphocytes population, CD4+ and CD8+ T cells were identified based on their binding to CD4 and CD8 antibodies, respectively

Human PBMCs were surface stained for CD20, CD4 and CD8 (Fig. 7) followed by intracellular staining for cyclins and CDKs. Cells were first permeabilized with 0.25% Triton X-100 in flow staining/washing buffer, vortexed and incubated 5 min at 4 °C. After washing twice in PBS, cells were stained for cyclins or CDK2. After 30 min incubation at room temperature, cells were washed twice and incubated with rabbit Alexa Fluor 647 secondary antibody (1:1000) for 30 min at 4 °C. After the final wash, cells were resuspended in 100 μl PBS containing 5 μl 7AAD and 50 μg/ml RNaseA, incubated for 30 min at room temperature. The samples were kept at 4 °C until they could be analyzed using a flow cytometer.

Mouse splenocytes were surface stained for CD3, CD4 and CD8 followed by 7AAD staining. Flow cytometric analysis was performed with, LSRII (Becton–Dickinson, Mountain View, CA) and FlowJo software (Tree Star, Inc) was used for data analysis. The distribution of cells into phases of the cell cycle was carried out by FlowJo. Expression of cyclins and CDK2 was determined based upon percentage of positive cells compared to isotype controls. Simultaneous bivariate analysis of DNA content and proliferation-associated proteins was used to relate the expression of cyclin E and CDK2 to the particular phases of the cell cycle.

### Western blotting

T cells were lysed with CellLytic-M (Sigma-Aldrich) along with 1× Halt protease and phosphatase inhibitor cocktail (Thermo Scientific) for preparation of whole cell lysate after 24 h of treatment. Protein concentration was measured by BCA assay (Pierce, Rockford, IL). SDS-PAGE and western blot analysis was performed as described earlier^15^. The primary antibodies were diluted in 1× TBS/0.1% Tween 20 and used to detect to E2F1 (1:1000, Cell Signaling, Danvers, MA) and β-actin (1:2000, Santa Cruz Biotech). Proteins were detected using secondary antibody conjugated to peroxidase (1:5000, Thermo Scientific) for 1 h at room temperature and visualized by using the supersignal West-Femto chemluminescent substrate (Thermo Scientific), acquired on a G:Box Chemi HR16 (Syngene) gel documentation system.

### Drug administration and ELISA

Male C57/BL6 mice (4 weeks old, Jackson Labs) were maintained in sterile microisolator cages under pathogen-free conditions in accordance with institutional ethical guidelines for care of laboratory animals, National Institutes of Health (NIH) guidelines, and the Institutional Animal Care Use Committee. Mice were weight matched and randomly assigned to two groups. To simulate the pattern used in most recreational METH abusers, an escalating dosing regimen as previously described was used^35^. Briefly, METH (0.45–10 mg/kg) was administered subcutaneously at the nape of the neck over the course of 6 days until 10 mg/kg was achieved; thereafter, mice received daily injections of METH at 10 mg/kg until the experimental endpoint. Peripheral blood samples were collected by submandibular bleeding as described by Golde et al^36^. METH levels were measured by ELISA according to manufacturer’s instruction (Abnova, Walnut, CA). The METH levels detected in the peripheral blood samples ranged from 7.8 ± 3.5 pM/ml of plasma.

### Histopathology and image analyses

Spleen tissue were collected at days 14, 28 and 56, fixed in 4% phosphate-buffered paraformaldehyde, embedded in paraffin. Indirect immunofluorescence was performed on 5 μm-thick serial sections. Section were deparaffinized, rehydrated and antigen retrieval was performed all together using TRILOGY (Cell Marque, Rocklin, CA) reagent according to manufacturer’s instruction. As a marker for cell proliferation, sections were incubated with anti-Ki-67 primary antibody (1:200) overnight at 4 °C. After removal and rinsing of the primary antibodies, spleen sections were incubated with Alexa-fluor 488-conjugated goat anti-rabbit secondary antibody (1:500) at room temperature for 1 h. Spleen sections were then washed and cover slipped with ProLong Antifade plus DAPI. Visualization of immunofluoresence was performed using an Eclipse 80i microscope (Nikon), fitted with a CoolSnap-EZ digital camera (Photometrics, Tucson, AZ, USA) at 20×. Image were acquired using NIS Elements R (Nikon) imaging software. The number of positive Ki-67 cells were quantitated from five microscopic fields for each tissue section using ImageJ software (Version 10.2, NIH, USA).

### Statistical analysis

Results are represented as means ± SEM and *p* values <0.05 are considered significant. The data were analyzed using Prism (GraphPad, La Jolla, CA) and statistical significance for multiple comparisons was assessed by one-way ANOVA followed by Fisher’s least significant difference test for multiple comparisons, or by the Student’s t-test for paired observations.

## References

[1] Colfax G, Shoptaw S (2005). The methamphetamine epidemic: implications for HIV prevention and treatment. Curr. HIV/AIDS Rep..

[2] Martinez LR, Mihu MR, Gacser A, Santambrogio L, Nosanchuk JD (2009). Methamphetamine enhances histoplasmosis by immunosuppression of the host. J. Infect. Dis..

[3] Nair MP (2009). Methamphetamine enhances HIV-1 infectivity in monocyte derived dendritic cells. J. Neuroimmune Pharmacol..

[4] Saito M (2008). Effects of single or repeated administrations of methamphetamine on immune response in mice. Exp. Anim..

[5] Mahajan SD (2006). Methamphetamine modulates gene expression patterns in monocyte derived mature dendritic cells: implications for HIV-1 pathogenesis. Mol. Diagn. Ther..

[6] Gonzales R, Marinelli-Casey P, Shoptaw S, Ang A, Rawson RA (2006). Hepatitis C virus infection among methamphetamine-dependent individuals in outpatient treatment. J. Subst. Abus. Treat..

[7] Yu Q (2002). Chronic methamphetamine exposure alters immune function in normal and retrovirus-infected mice. Int. Immunopharmacol..

[8] Phillips TR, Billaud JN, Henriksen SJ (2000). Methamphetamine and HIV-1: potential interactions and the use of the FIV/cat model. J. Psychopharmacol..

[9] Zule WA, Desmond DP (1999). An ethnographic comparison of HIV risk behaviors among heroin and methamphetamine injectors. Am. J. Drug. Alcohol Abus..

[10] Iwasa H, Kikuchi S, Hasegawa S, Suzuki K, Sato T (1996). Alteration of G protein subclass mRNAs in methamphetamine-induced behavioral sensitization. Ann. N Y Acad. Sci..

[11] Sriram U (2016). Methamphetamine induces trace amine-associated receptor 1 (TAAR1) expression in human T lymphocytes: role in immunomodulation. J. Leukoc. Biol..

[12] Talloczy Z (2008). Methamphetamine inhibits antigen processing, presentation, and phagocytosis. PLoS. Pathog..

[13] Prlic M, Williams MA, Bevan MJ (2007). Requirements for CD8 T-cell priming, memory generation and maintenance. Curr. Opin. Immunol..

[14] Harms R, Morsey B, Boyer CW, Fox HS, Sarvetnick N (2012). Methamphetamine administration targets multiple immune subsets and induces phenotypic alterations suggestive of immunosuppression. PLoS. ONE.

[15] Potula R (2010). Methamphetamine causes mitrochondrial oxidative damage in human T lymphocytes leading to functional impairment. J. Immunol..

[16] Sherr CJ, Roberts JM (1999). CDK inhibitors: positive and negative regulators of G1-phase progression. Genes. Dev..

[17] Sherr CJ (2000). The Pezcoller lecture: cancer cell cycles revisited. Cancer Res..

[18] Scholzen T, Gerdes J (2000). The Ki-67 protein: from the known and the unknown. J. Cell. Physiol..

[19] In SW, Son EW, Rhee DK, Pyo S (2004). Modulation of murine macrophage function by methamphetamine. J. Toxicol. Environ. Health A.

[20] Sriram U (2016). Impaired subset progression and polyfunctionality of T cells in mice exposed to methamphetamine during chronic LCMV infection. PLoS. ONE.

[21] Sriram U, Haldar B, Cenna JM, Gofman L, Potula R (2015). Methamphetamine mediates immune dysregulation in a murine model of chronic viral infection. Front. Microbiol..

[22] Fisher D, Gamieldien K, Mafunda PS (2015). Methamphetamine is not toxic but disrupts the cell cycle of blood-brain barrier endothelial cells. Neurotox. Res..

[23] Jackson AR, Shah A, Kumar A (2014). Methamphetamine alters the normal progression by inducing cell cycle arrest in astrocytes. PLoS. ONE.

[24] Pardee AB (1989). G1 events and regulation of cell proliferation. Science.

[25] Bertoli C, Skotheim JM, de Bruin RA (2013). Control of cell cycle transcription during G1 and S phases. Nat. Rev. Mol. Cell. Biol..

[26] Geisen C, Moroy T (2002). The oncogenic activity of cyclin E is not confined to Cdk2 activation alone but relies on several other, distinct functions of the protein. J. Biol. Chem..

[27] Weinberg RA (1995). The retinoblastoma protein and cell cycle control. Cell.

[28] Bartek J, Bartkova J, Lukas J (1996). The retinoblastoma protein pathway and the restriction point. Curr. Opin. Cell. Biol..

[29] Zarkowska T, Mittnacht S (1997). Differential phosphorylation of the retinoblastoma protein by G1/S cyclin-dependent kinases. J. Biol. Chem..

[30] Dick FA, Rubin SM (2013). Molecular mechanisms underlying RB protein function. Nat. Rev. Mol. Cell. Biol..

[31] Schluter C (1993). The cell proliferation-associated antigen of antibody Ki-67: a very large, ubiquitous nuclear protein with numerous repeated elements, representing a new kind of cell cycle-maintaining proteins. J. Cell. Biol..

[32] Weitkamp JH, Crowe JE (2001). Blood donor leukocyte reduction filters as a source of human B lymphocytes. Biotechniques.

[33] Neron S, Dussault N, Racine C (2006). Whole-blood leukoreduction filters are a source for cryopreserved cells for phenotypic and functional investigations on peripheral blood lymphocytes. Transfusion.

[34] Neron S (2007). Characterization of mononuclear cells remaining in the leukoreduction system chambers of apheresis instruments after routine platelet collection: a new source of viable human blood cells. Transfusion.

[35] Ramirez SH (2009). Methamphetamine disrupts blood-brain barrier function by induction of oxidative stress in brain endothelial cells. J. Cereb. Blood Flow. Metab..

[36] Golde WT, Gollobin P, Rodriguez LL (2005). A rapid, simple, and humane method for submandibular bleeding of mice using a lancet. Lab. Anim..

